# Knockdown of *vATPase subunit A* impairs larval-pupal development in *Plutella xylostella*

**DOI:** 10.3389/fphys.2026.1974591

**Published:** 2026-10-09

**Authors:** Xuetao Yu, Yanan Jiang, Lijie Ren, Jinhua Luo, Zhanhao Dai, Lin Lu, Li Ren, Li Zhu, Chao Li, Qidi Wei, Gang Wu, Xiaohong Yan, Chenhui Shen

**Affiliations:** 1 Oil Crops Research Institute of Chinese Academy of Agricultural Sciences, Ministry of Agriculture and Rural Affairs, Wuhan, China; 2 Key Laboratory of Biology and Genetic Improvement of Oil Crops, Xining, Plant Protection of Observation and Research Station, Xining, China; 3 Yazhouwan National Laboratory, Sanya, China; 4 Enshi Tujia and Miao Autonomous Prefecture Academy of Agricultural Sciences, Enshi, China; 5 Guizhou Institute of Crop Germplasm Resources, Guiyang, China

**Keywords:** development, microinjection, *Plutella xylostella*, *PxvATPaseA*, RNA interference

## Abstract

*Plutella xylostella* is a destructive pest damaging the cruciferous plants worldwide. Transgenic plants expressing dsRNA can be widely deployed for pest control. However, highly effective lethal target genes are currently lacking. Here, we assessed the efficacy of RNA interference (RNAi) against *P. xylostella* through microinjection of *in vitro* synthesized dsRNA targeting *PxvATPaseA*. The full-length ORF of *PxvATPaseA* was 1848 bp, encoding 615 amino acids. *PxvATPaseA* was widely transcribed during different developmental stages from egg to adult. It was abundantly expressed in the foregut, hindgut, and Malpighian tubules, compared with other tissue types. Injecting 800 ng of ds*PxvATPaseA* into the fourth-instar larvae greatly reduced the corresponding mRNA level by 1.88-fold on day 2, causing 45.8% larval mortality, and lastly 54.2% treated larvae successfully pupated. At a higher dose of 1200 ng of ds*PxvATPaseA*, RNAi efficacy was significantly enhanced. The expression level of *PxvATPaseA was* reduced by 2.22-fold and 1.92-fold on day 2 and 3, respectively, leading to 79.2% larval lethality, and only 20.8% of depleted larvae pupated. Our results indicate that *PxvATPaseA* is an effective target for managing *P. xylostella*. These findings will facilitate the development of new insecticides and the cultivation of pest-resistant plants.

## Introduction

The diamondback moth, *Plutella xylostella*, represents one of the most economically devastating pests affecting cruciferous crops, causing an estimated annual loss of US $770 million in China and US $4–5 billion globally (Shehzad et al., 2023; Yin et al., 2025). Its biological characteristics enable rapid evolution of resistance, with documented tolerance to over 100 chemical pesticides (Shehzad et al., 2023). Therefore, developing novel control strategies for this pest is urgently needed.

RNA interference (RNAi) is a conserved cellular regulatory pathway that utilizes double-stranded RNA (double-stranded RNA, dsRNA) to mediate precise, sequence-dependent gene silencing, which has garnered significant attention as a promising approach for sustainable pest management (Zhang et al., 2015, 2017; Li et al., 2022a). Recent studies have shown transgenic plants expressing dsRNA can be effectively deployed for pest management, which was classified into two distinct categories: categories nuclear transformation (NT) lines and plastid transformation (PT) lines (Zhang et al., 2015, 2017; Li et al., 2022a). For example, applying transgenic plant *Nicotiana tabacum* expressing dsRNA of different genes to manage *Helicoverpa armigera* (Xiong et al., 2013; Mamta et al., 2016), *Myzus persicae* (Zhao et al., 2016; Rauf et al., 2019), *Bemisia tabaci* (Dong et al., 2020), *Chilo partellus*, *P. xylostella* and *Maruca vitrata* (Rana et al., 2020). Furthermore, numerous transgenic plants, including *Arabidopsis thaliana* (Bhatia and Bhattacharya, 2018; Eakteiman et al., 2018), *Gossypium hirsutum* (Han et al., 2017), *Oryza sativa* (Yang et al., 2021), *Glycine max* (Dubey et al., 2017; Meng et al., 2017), *Zea mays* (Baum et al., 2007), *Solanum tuberosum* (Hussain et al., 2019; Naqqash et al., 2020), *Lactuca sativa* (Ibrahim et al., 2017), *Brassica napus* (Deng et al., 2024) are generated to control pests. Notably, transgenic *B. napus* producing *dsCHS1* significantly reduced larval weight and increased mortality in *P. xylostella* (Deng et al., 2024). These findings demonstrate the feasibility of using dsRNA-expressing transgenic plants to manage *P. xylostella* populations. Nonetheless, the identification of effective molecular targets capable of inducing high lethality in pests stands as a fundamental requirement for the successful deployment of this transgenic technology.

Vacuolar-type H^+^-ATPase (V-ATPase) holoenzyme represents an evolutionarily conserved multisubunit complex ubiquitous across eukaryotic organisms. Its core function involves the hydrolysis of adenosine triphosphate (ATP), resulting in the formation of adenosine diphosphate (ADP) and inorganic phosphate, while concurrently translocating protons across cellular membranes (Zeng et al., 2021a, b, 2022). In terms of architecture, the V-ATPase holoenzyme exhibits a bipartite structural organization consisting of the cytosolic V1 sector (encompassing eight subunits A to H) that drives ATP breakdown, and the membrane-embedded V0 sector (consisting of five subnits a_1_d_1_c_4-5_c’_1_c”_1_) that facilitates proton translocation across cellular membranes (Mazhab-Jafari et al., 2016; McGuire et al., 2017; Roh et al., 2018; Abbas et al., 2020; Song et al., 2020).

Previous research has identified vATPaseA as an attractive candidate target for RNAi-mediated pest control. Silencing *vATPaseA* in *Hyphantria cunea* impaired intestinal development and increased larval mortality (Wang et al., 2024). In *Plagiodera versicolora*, knockdown of *vATPase-A* resulted in high mortality rates in both larvae and adults (Li et al., 2022b). Similar lethal effects have been observed in *Acalymma vittatum* (Kaplanoglu et al., 2026), *Manduca sexta* (Burke et al., 2019), *Bemisia tabaci* (Thakur et al., 2014) and *H. armigera* (Sharif et al., 2022). Collectively, these studies suggest that vATPaseA may serve as an effective target for controlling *P. xylostella* via RNAi.

In this study, we investigated the biological function of *vATPaseA* by assessing the impacts of two doses of ds*vATPaseA* by microinjection in *P. xylostella*. Our findings identify *PxvATPaseA* as a potential to aid in the development of new insecticides and the cultivation of pest-resistant plants.

## Methods and materials

### Insect rearing

*P. xylostella* was collected from Brassica napus in Haidong City, Qinghai Province, China and raised on Wuhan City, Hubei Province, China. The larvae of *P. xylostella* were fed on excised leaves of *B. napus* under precise experimental conditions (Temperature: 26 ± 1 °C; Relative humidity: 60% ± 5%; Photoperiod: 16-hour light:8-hour dark). The adults were supplied with a 10% honey solution for nutrition.

### Molecular cloning

The putative *PxvATPaseA* sequence was required from publicly available transcriptome and genome datasets of *P. xylostella* (Xie et al., 2022; Niu et al., 2025). To ensure sequence correctness, we performed PCR using 2 × Phanta™ Master Mix (Vazyme, Nanjing, China), based on gene-specific primers (**Supplementary Table S1**, **Supplementary Figure S1**). The calculated molecular weight and isoelectric point were calculated by the Compute pI/MW tool (https://web.expasy.org/compute_pi/). The accession number of full-length cDNA sequence was ON108624 (GenBank). Phylogenetic construction was carried out using MEGA-5 software (https://sourceforge.net/projects/mega5/), applying the neighbor-joining method with 1,000 bootstrap replications.

### DsRNA synthesis

Two cDNA fragments corresponding to *PxvATPaseA* and enhanced green fluorescent protein, *GFP*) were PCR-amplified via gene-specific primers (**Supplementary Table S1**). To minimize off-target effects, we performed BLASTN analysis of target sequences against the *P. xylostella* transcriptome database, excluding regions with ≥20 bp contiguous homology to non-target genes. dsRNA was generated *in vitro* using the MEGAscript T7 High Yield Transcription Kit (Ambion, Austin, TX, USA) following the manufacturer’s protocol. Purified dsRNA concentrations were quantified by agarose gel electrophoresis and spectrophotometry (Nanodrop 1000). Aliquots were preserved at -80 °C until experimental application.

### DsRNA microinjection

DsRNA microinjection was performed as described previously (Yu et al., 2025, 2026). Briefly, newly ecdysed fourth-instar larvae were injected with either 800 ng or 1200 ng of ds*PxvATPaseA*. The individuals injected with ds*GFP* were used as negative controls. Each treatment group consisted of 8 larvae per replicate, with 6 biological replicates per dose. At 2 and 3 days post-injection, three replicates were processed for qRT-PCR analysis to measure the efficiency of RNA interference. The remaining three replicates were observed daily for three weeks to record survival rate, weight changes, pupation and adult emergence rate. The defect phenotypes were photographed through a stereoscope MZ62 (Mingmei, Guangzhou, China).

### Real-time quantitative PCR

Total RNA was isolated from all experimental samples to analyze temporal transcription patterns (eight individuals pooled per sample) and tissue-specific expression profiles (30 tissues pooled per sample). For treatment group analysis, total RNA was extracted using TRIzol reagent (YiFeiXue Tech, Nanjing, China), with each of samples containing 8 pooled larvae and three biological replicates. qRT-PCR was performed to measure the relative abundance using previously described protocols (Wang et al., 2024). The housekeeping gene ribosomal protein *RPL32 (PxRPL13)* was selected as the internal control. Each qRT-PCR assay included three biological replicates and three technical replicates to ensure reproducibility. Relative mRNA transcripts were computed via the 2^-ΔΔCt^ method.

### Data analysis

Statistical analyses were carried out using SPSS version 26.0 for Windows (IBM Corp., Chicago, IL, USA). Data are presented as mean ± standard error (SE), and significant differences among treatment groups were determined using the Tukey-Kramer multiple comparison test. Survival curves were analyzed using GraphPad Prism version 8.0 with the log-rank (Mantel-Cox) test (95% confidence interval).

## Results

### Identification of *PxvATPaseA*

A putative full-length *PxvATPaseA* was obtained in *P. xylostella* by mining the transcriptome and genome data. The full-length ORF of *PxvATPaseA* was 1848 bp, encoding 615 amino acids with a predicted molecular weight of 68.12 kDa and theoretical pI of 5.12. Domain analysis showed that *Px*vATPaseA contained a conserved V-ATPase_V1_A domain (**Figure 1A**).

**Figure 1:**
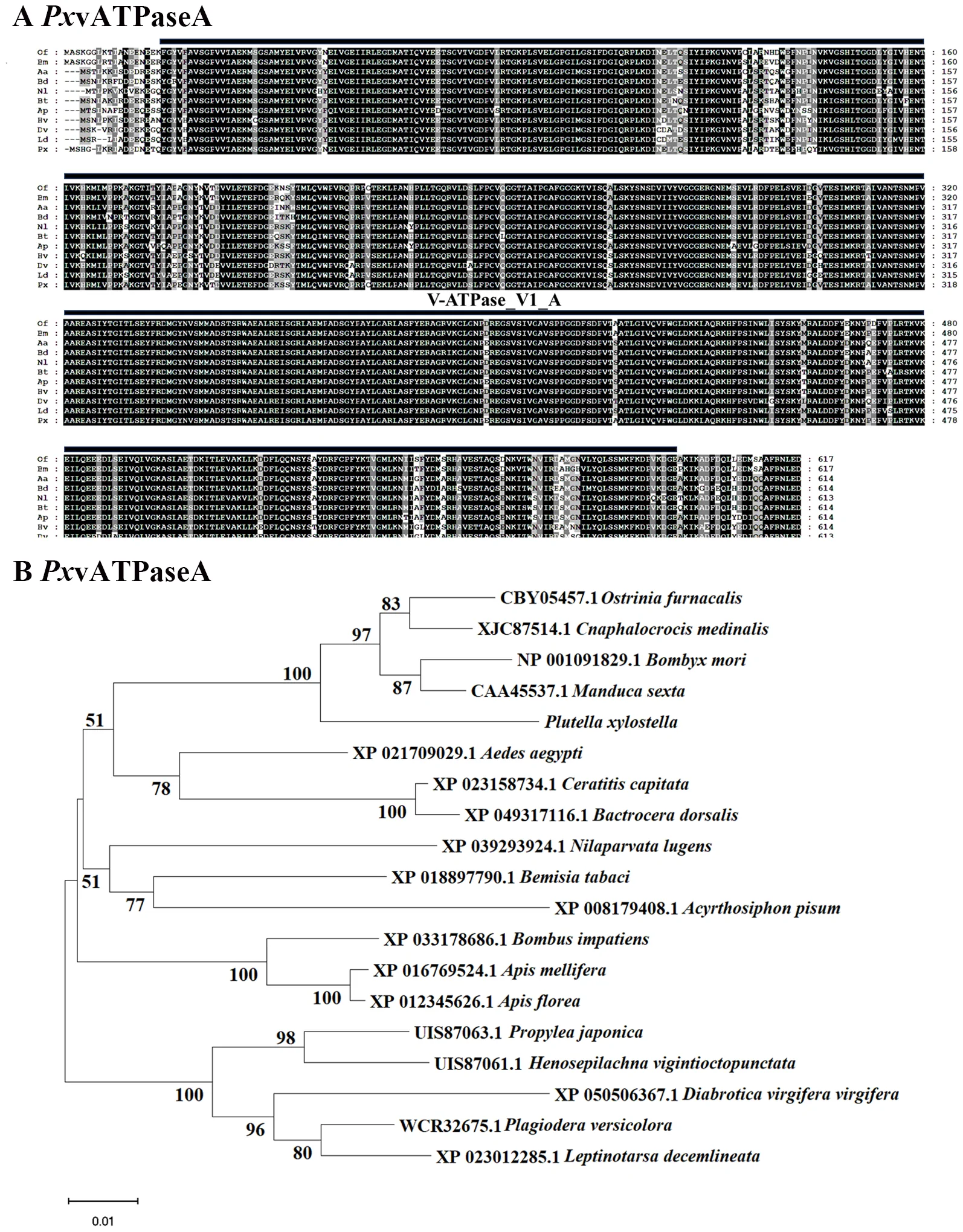
Alignment **(A)** and phylogenetic analysis **(B)** of vATPase subunits A (*PxvATPaseA*) from *Plutella xylostella*. **(A)** The vATPaseA protein sequences were obtained from *Ostrinia furnacalis*, *Bombyx mori*, *Aedes aegypti*, *Bactrocera dorsalis*, *Nilaparvata lugens*, *Bemisia tabaci*, *Acyrthosiphon pisum*, *Henosepilachna vigintioctopunctata*, *Diabrotica virgifera virgifera*, *Leptinotarsa decemlineata*, and *Plutella xylostella*. The V-ATPase_V1_A domain is indicated by a black line. **(B)** The amino acid sequences of the *PxvATPaseA* subunit were derived from five lepidopteran species: *Ostrinia furnacalis*, *Manduca sexta*, *Cnaphalocrocis medinalis*, *Bombyx mori*, and *Plutella xylostella*; three dipteran species: *Aedes aegypti*, *Ceratitis capitata*, and *Bactrocera dorsalis*; three hemipteran species: *Nilaparvata lugens*, *Bemisia tabaci*, and *Acyrthosiphon pisum*; three hymenopteran species: *Apis mellifera*, *Apis florea*, and *Bombus impatiens*; and five coleopteran species: Propylea japonica, *Henosepilachna vigintioctopunctata*, *Diabrotica virgifera virgifera*, *Plagiodera versicolora* and *Leptinotarsa decemlineata*. bootstrap analyses were performed with 1000 replications, and bootstrap values exceeding 50% are indicated on the tree.

Phylogenetic analysis of vATPaseA from 19 insect species was built to assess the evolutionary relationships (**Figure 1B**).These sequences were from five lepidoptera: *Ostrinia furnacalis*, *M. sexta*, *Cnaphalocrocis medinalis*, *Bombyx mori*, and *P. xylostella*; three diptera: *Aedes aegypti*, *Ceratitis capitata*, and *Bactrocera dorsalis*; three hemiptera: *Nilaparvata lugens*, *B. tabaci*, and *Acyrthosiphon pisum*; three hymenoptera: *Apis mellifera*, *Apis florea*, and *Bombus impatiens*; and five coleoptera: *Propylea japonica*, *Henosepilachna vigintioctopunctata*, *Diabrotica virgifera virgifera*, *P. versicolora*, and *Leptinotarsa decemlineata*. The analysis included species from five insect orders: Lepidoptera, Diptera, Hemiptera, Hymenoptera and Coleoptera. The resulting unrooted tree revealed that *Px*vATPaseA clustered within the Lepidoptera subclade (**Figure 1B**).

### Expression analysis of *PxvATPaseA*

qRT-PCR was employed to analyze the temporal expression profile of the *PxvATPaseA*. *PxvATPaseA* was broadly expressed across all developmental stages. *PxvATPaseA* mRNA expression was significantly elevated in the early stage of each larval stage (second-, third- and fourth-instar larvae), and low at day 3 of the fourth-instar larvae (**Figure 2A**).

**Figure 2:**
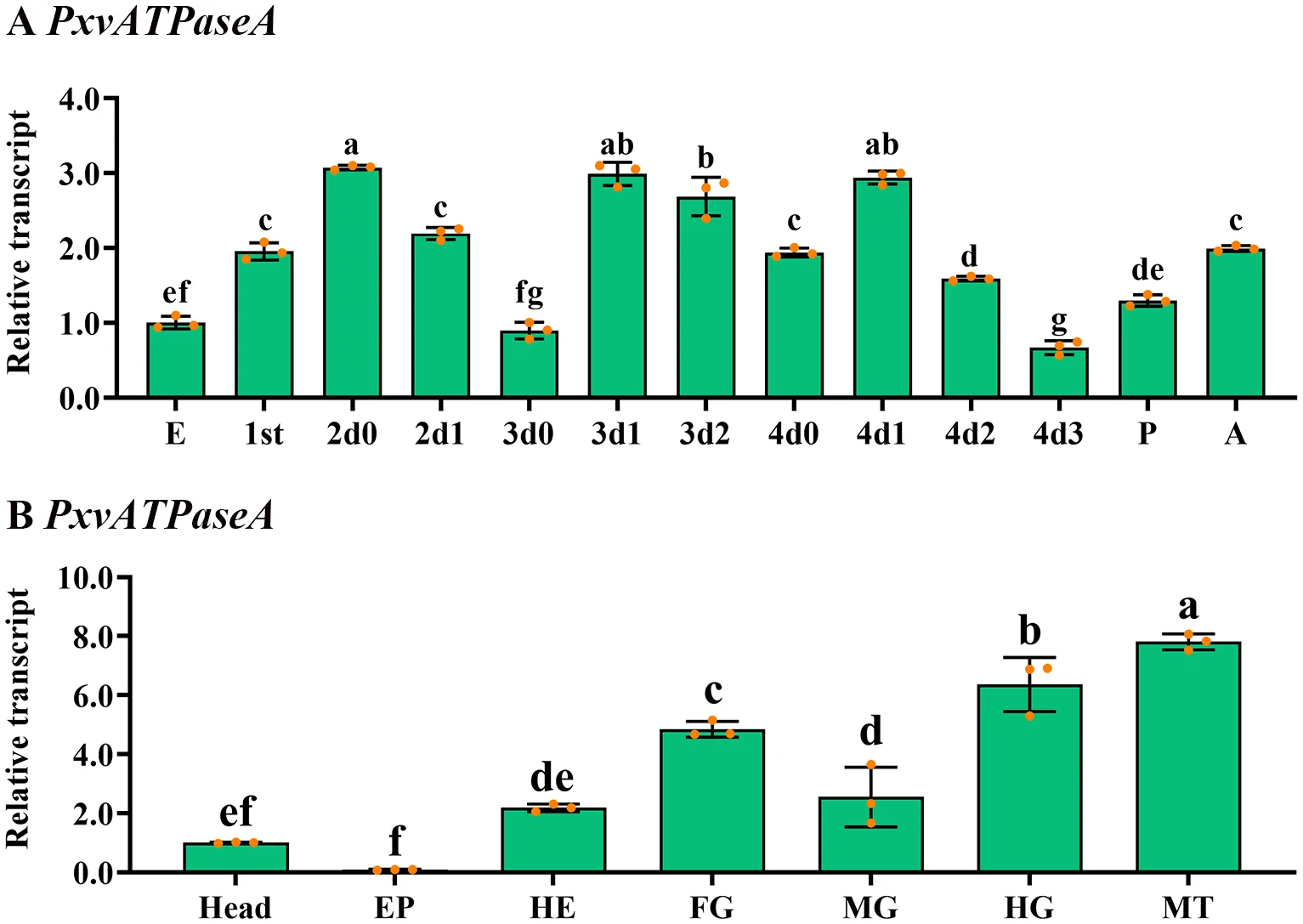
Temporal **(A)** and Tissue **(B)** expression patterns of *PxvATPaseA* in *Plutella xylostella*. **(A)** For analyzing temporal transcription profiles, cDNA templates were prepared from eggs, larvae of the first to fourth instars collected at daily intervals (for first instar: 0–1 day after hatching; for other instars: day 0 refers to newly molted individuals), as well as newly ecdysed pupae and newly emerged adults. **(B)** For tissue-specific expression patterns, templates were derived from the head, foregut (FG), midgut (MG), hindgut (HG), hemolymph (HE), Malpighian tubules (MT), and epidermis (EP) of day-4 fourth-instar larvae. Each sample contained three independent biological replicates, with each including 5–10 individuals, and all qRT-PCR reactions were performed in technical triplicates. Expression levels were calculated using the 2^−ΔΔCT^ method. Data are presented as column means with vertical bars indicating the standard error (SE).

The tissue-specific expression pattern of PxvATPaseA was systematically analyzed across all examined tissues, including the head, foregut, midgut, hindgut, hemolymph, Malpighian tubules, and epidermis. The results indicated that *PxvATPaseA* was constitutively expressed in various tissues, with the highest expression observed in the foregut, hindgut, and Malpighian tubules, followed by moderate levels in the head, hemolymph, and midgut, and the lowest expression in the epidermis (**Figure 2B**).

### Impacts of injecting 800 ng ds*PxvATPaseA* into fourth-instar larvae

To assess the effects of *PxvATPaseA*-targeted RNAi in *P. xylostella*, we performed gene silencing using dsRNA microinjection. Microinjection of 800 ng ds*PxvATPaseA* into newly-molted fourth-instar larvae resulted in a significant 46.8% reduction in *PxvATPaseA* mRNA levels by day 2 post-injection, but no significant difference was observed on day 3 (**Figure 3A**).

**Figure 3:**
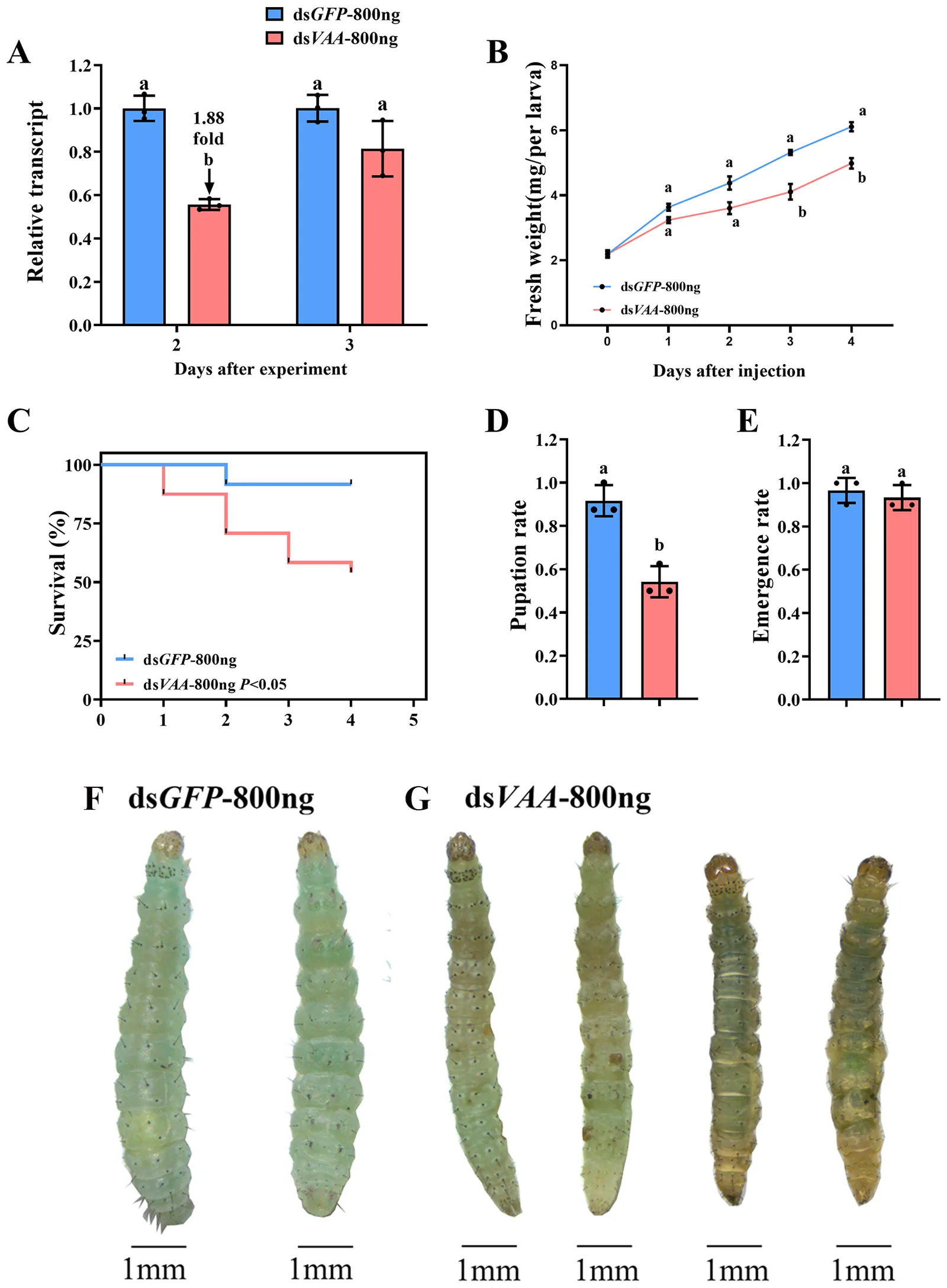
Impacts on RNAi of *PxvATPaseA* in fourth-instar larvae in *Plutella xylostella*. Newly ecdysed fourth-instar larvae were microinjected with 0.2 µL of solution containing 800 ng of ds*PxvATPaseA*, with an equivalent amount of ds*GFP* as the control. *PxvATPaseA* transcript abundance were quantified at day 2 and 3 post-injection **(A)**. Relative mRNA levels are presented as the ratio of relative copy numbers in treated larvae to those in the ds*GFP* control group (set as 1). Larval weight **(B)**, survival rate **(C)**, pupation rate **(D)**, and emergence rate **(E)** were recorded over a 5-day trial period. Data are presented as mean ± SE. Different letters denote statistically significant differences at *P* < 0.05. The defective phenotypes of larvae that have been injected ds*PxvATPaseA* are shown **(G)**, compared with those in controls **(F)**.

Knockdown of *PxvATPaseA* significantly inhibited larval development, with fresh weights lowered by 22.6% and 18.5% on day 3 and 4, respectively (**Figure 3B**). RNAi-mediated silencing of *PxvATPaseA* resulted in high larval mortality, with approximately 41.7% and 45.8% of treated larvae dying on days 3 and 4 post-injection, respectively, and the mortality rate of larvae in the ds*GFP* group was 8.33% (**Figure 3C**). Silencing *PxvATPaseA* disrupted larval-pupal development, resulting in successful pupation of only 54.2% of treated larvae (**Figure 3D**). In contrast, knockdown of *PxvATPaseA* had no significant effect on adult emergence rates (**Figure 3E**). Larvae treated with ds*PxvATPaseA* exhibited small and misshapen body size, gradually darkening and lastly dying (**Figures 3F vs. 3G**).

### Effects of injecting 1200 ng ds*PxvATPaseA* into fourth-instar larvae

Injection of 1200 ng ds*PxvATPaseA* greatly lowered the *PxvATPaseA* transcript by 55.0% and 47.9% on days 2 and 3, respectively, compared with ds*GFP*-injected controls (**Figure 4A**).

**Figure 4:**
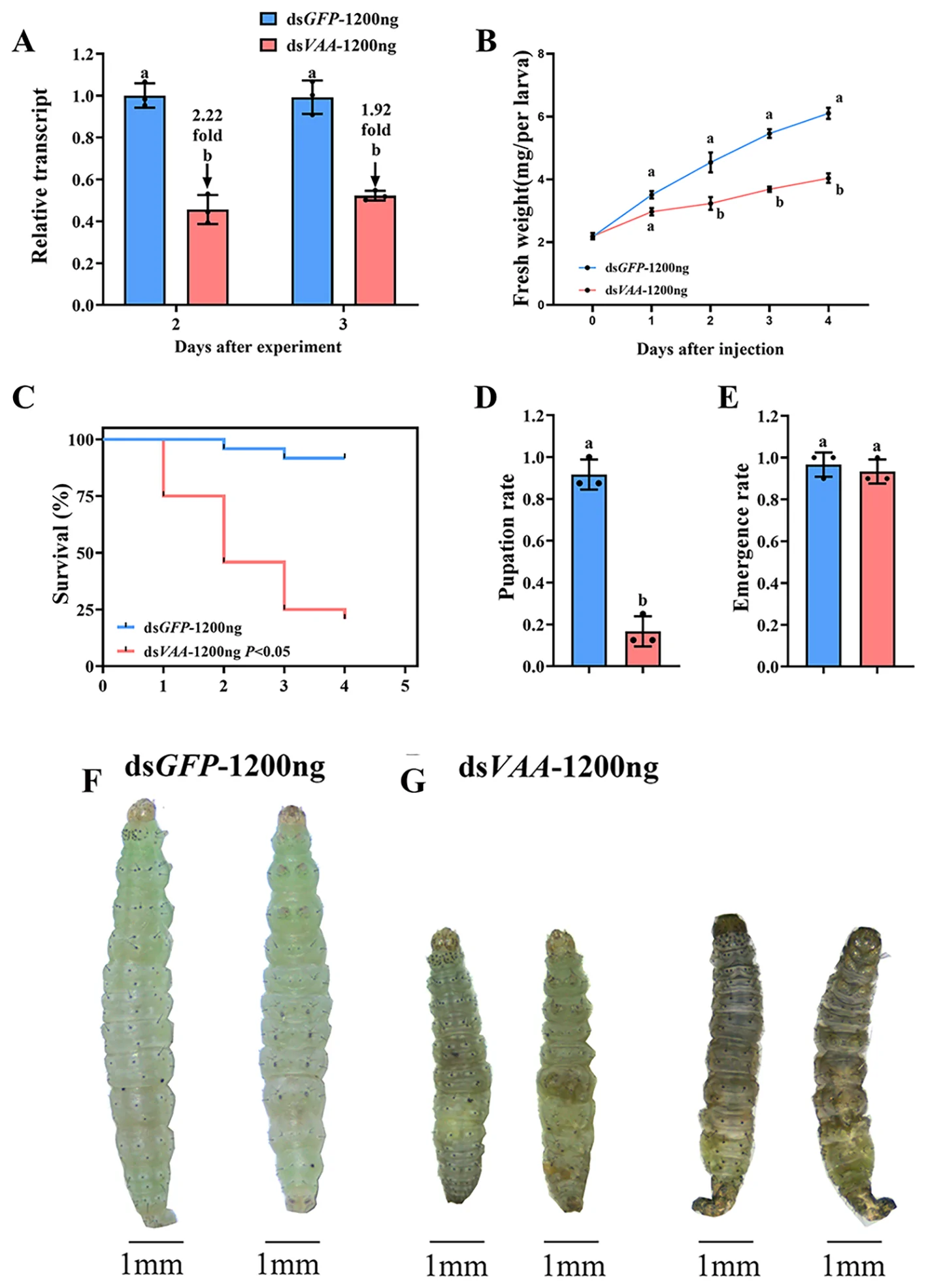
Effects of silencing of *PxvATPaseA* on the fourth-instar larvae of *Plutella xylostella*. Newly ecdysed fourth-instar larvae were administered with 0.2 µL of solution containing 1200 ng of ds*PxvATPaseA*, with ds*GFP* injection used as the negative control. Under identical experimental conditions, *PxvATPaseA* transcripts **(A)**, larval weight **(B)**, survival rate **(C)**, pupation **(D)** and emergence rate **(E)** were analyzed. Data are shown as mean ± SE. Different letters indicate statistically significant differences at *P* < 0.05. The defective phenotypes of larvae that have been injected ds*PxvATPaseA* are shown **(G)**, compared with those in controls **(F)**.

Knockdown of *PxvATPaseA* exerted a potent inhibitory effect on larval growth, resulting in significant reductions in fresh weight of 28.9%, 32.8%, and 33.8% on days 2, 3, and 4 post-injection, respectively (**Figure 4B**). RNAi of *PxvATPaseA* led to larval mortality, reaching 54.8%, 75.0% and 79.2% on day 2, 3 and 4, respectively (**Figure 4C**). Ultimately, only 20.8% of ds*PxvATPaseA*-treated larvae pupated (**Figure 4D**), though the adult emergence rate did not differ significantly between treatment and control groups (**Figure 4E**). *PxvATPaseA*-silenced larvae exhibited a complete failure to undergo pupal ecdysis, displaying stunted growth and abnormal body morphology, compared to ds*GFP*-injected controls. These *PxvATPaseA*-treated hypomorphs gradually became withered, dried and blackened, and eventually died (**Figures 4F vs. 4G**).

## Discussion

vATPase is a multi-subunit complex involved in various biological functions across living organisms (Zeng et al., 2021c). To date, the function of vATPaseA in *P. xylostella* has not been characterized. In this study, the sequence of *vATPaseA* in *P. xylostella* was identified from the transcriptome and genome data. RNAi-mediated silencing of *PxvATPaseA* via microinjection of two distinct dsRNA doses inhibited larval development, impaired pupae and ultimately caused high larval mortality. These results support that *PxvATPaseA* is an effective molecular target for sustainable management of *P. xylostella* larvae.

Domain analysis indicated the *Px*vATPaseA protein is highly conserved across species, containing a V-ATPase_V1_A domain (**Figure 1**). Additionally, a phylogenetic tree constructed from vATPaseA amino acid sequences of 19 insect species revealed that *Px*vATPaseA clusters within the Lepidoptera subclade (**Figure 1**). These results highlight the potential of targeting *vATPaseA* as an alternative RNAi-based strategy for managing *P. xylostella*.

*PxvATPaseA* was broadly expressed throughout all developmental stages, from embryo (egg) to adult. The mRNA level of *PxvATPaseA* was high in the early stage of each larval stage (second-, third- and fourth-instar larvae), and low at day 3 of fourth-instar larvae (**Figure 2**). Comparatively, peak vATPaseA expression in *H. cunea* occurs in third-instar larvae (Wang et al., 2024), whereas in *P. versicolora*, the highest expression is observed in first-instar larvae (Li et al., 2022b). The elevated mRNA levels of *PxvATPaseA* during larval stages suggest a critical role larval growth and development in *P. xylostella*.

Tissue-specific expression patterns indicated that *PxvATPaseA* was highly expressed in the foregut, hindgut and Malpighian tubules (**Figure 2**). Consistent with our findings, in *H. vigintioctopunctata*, the mRNA level of *vATPaseA* was highest in the Malpighian tubules (Guo et al., 2021), while in *P. versicolora, vATPaseA* was highly transcribed in the hindgut and Malpighian tubules (Li et al., 2022b). These similar expression profiles indicate that vATPaseA serves a vital function in toxin metabolism and waste excretion (Sharif et al., 2022).

Although studies have shown that RNAi targeting *PxTH* (Hou et al., 2024), *PxCHS1* (Deng et al., 2024), *PxvATPasea* that has an important function inion homeostasis (Yu et al., 2025), *PxvATPaseE* that underscores a critical role in nutrient absorption (Yu et al., 2026) induced high mortality in *P. xylostella*, lepidopteran pests can evolve resistance to RNAi through various mechanisms, including dsRNA instability, restricted systemic spread, endosomal entrapment, and dysfunction of core RNAi machinery (Cooper et al., 2019). Double-stranded ribonucleases (dsRNases), present in insect hemolymph and gut fluid, are widely recognized as a key barrier to RNA interference (RNAi) effectiveness (Cooper et al., 2019). By targeting dsRNA molecules circulating in bodily fluids, these enzymes compromise dsRNA stability, thereby impeding RNAi performance. Inhibiting dsRNase activity in insects can mitigate intracellular dsRNA breakdown and significantly boost RNAi efficiency (Yu et al., 2025). In additions, Encapsulating double-stranded RNA (dsRNA) within protective nanomaterials serves as an innovative delivery strategy for RNA interference (RNAi) applications, effectively preserving the structural integrity of dsRNA molecules. When combined with targeted inhibition of double-stranded ribonucleases (dsRNases), this approach minimizes intracellular dsRNA degradation and significantly enhances RNAi efficacy. These nanocarrier complexes exhibit high transduction efficiency and low cytotoxicity, facilitating efficient cellular uptake of dsRNA while shielding it from nuclease-mediated degradation under fluctuating environmental conditions, such as variable temperatures and dynamic medium compositions (Yu et al., 2026).Therefore, identifying highly effective lethal target genes is essential for sustainable control of lepidopteran pests such as *P. xylostella*.

Our results demonstrated that microinjection of ds*PxvATPaseA* at two doses significantly suppressed *PxvATPaseA* mRNA levels, reduced larval fresh weight, and caused high mortality (**Figures 3**, **4**). Consistent with previous studies, knockdown of *vATPaseA* in *H. cunea* (Wang et al., 2024), *H. vigintioctopunctata* (Guo et al., 2021) and *P. versicoloraa* (Li et al., 2022b) resulted in significant lethal effects. Furthermore, transgenic *B. napus* expressing *hpPxCHS1* increased larval mortality (Deng et al., 2024), suggesting that *PxvATPaseA* could similarly serve as an effective target for developing genetically modified crops to control *P. xylostella.*

## Data Availability

The original contributions presented in the study are included in the article/**Supplementary Material**. Further inquiries can be directed to the corresponding authors.
