# Supplementary material for: Knockdown of vATPase subunit A impairs larval-pupal development in Plutella xylostella
Source: Front Physiol. 2026 Oct 9;17:1974591. doi: 10.3389/fphys.2026.1974591 (PMC13690533; doi:10.3389/fphys.2026.1974591)
Supplement: Supplementary file 1 [file Table1.doc]

Supplementary data

**Knockdown of *vATPase subunits A* impairs larval-pupal development in *Plutella xylostella***

Xuetao Yu^1†^, Yanan Jiang^2†^, Lijie Ren^1^, Jinhua Luo^3^, Zhanhao Dai^1^, Lin Lu^1^, Li Ren^1^, Li Zhu^1^, Chao Li^1^, Qidi Wei^4^, Gang Wu^1^, Xiaohong Yan^1^*, Chenhui Shen^1^*

1. Key Laboratory of Biology and Genetic Improvement of Oil Crops, Xining，Plant Protection of Observation and Research Station, Oil Crops Research Institute of Chinese Academy of Agricultural Sciences, Ministry of Agriculture and Rural Affairs, Wuhan 430062, China
2. Yazhouwan National Laboratory, Sanya 572025, China
3. Enshi Tujia and Miao Autonomous Prefecture Academy of Agricultural Sciences,Enshi 445000,China
4. Guizhou Institute of Crop Germplasm Resources, Guiyang 550006, China

**Table S1. Primers used in RT-PCR, dsRNA synthesis and qPCR**

| **Fragment name** | **Forward primer** | | **Reverse primer** |
| --- | --- | --- | --- |
| **RT-PCR** |  | |  |
| *PxvATPaseA* | ATGAGTCACGGGTTGAAGAGGA | | TTAGTCCTCTAGGTTACGGAAGGC |
| **dsRNA synthesis** |  | |  |
| ds*vATPaseA* | GGACGACTTCTACGACAAGAACTACC | | TCTTTCACGGGGTCCTTGAA |
| ds*GFP* | TAATACGACTCACTATAGG | | CCTATAGTGAGTCGTATTA |
| **qPCR** |  | |  |
| q*PxvATPaseA* | GGGCTACAACGTGTCCATGA | | GACACTTGACTCTCCCAGCC |
| q*PxRPL32* | CCAATTTACCGCCCTACC | | TACCCTGTTGTCAATACCTCT |
|  | |  |  |
